# Impact of Hysteroscopic Instillation of Autologous Platelet-Rich Plasma on Pregnancy Outcomes in Patient With Recurrent Implantation Failure: A Case Report

**DOI:** 10.7759/cureus.68449

**Published:** 2024-09-02

**Authors:** Pavan Tej, Akash More, Avanti Kalbande, Nancy Nair

**Affiliations:** 1 Clinical Embryology, Datta Meghe Medical College, Datta Meghe Institute of Higher Education and Research, Nagpur, IND; 2 Clinical Embryology, Datta Meghe Institute of Higher Education and Research, Wardha, IND; 3 Obstetrics and Gynaecology, Shalinitai Meghe Hospital and Research Centre, Datta Meghe Institute of Higher Education and Research, Nagpur, IND

**Keywords:** intrauterine insemination (iui), intracytoplasmic sperm injection (icsi), assisted reproductive technology (art), hysteroscopy, platelet-rich plasma (prp), recurrent implantation failure (rif)

## Abstract

Recent advancements in assisted reproductive technology (ART) have enabled couples to achieve pregnancy, who were previously unable to conceive. However, recurrent implantation failure (RIF) remains a significant challenge. This case study exhibits the effective use of hysteroscopic-guided platelet-rich plasma (PRP) instillation in the treatment of a female patient aged 33 who was nulliparous and diagnosed with RIF and a thin endometrium, which resulted in primary infertility. The couple had a history of 10 years of infertility and had previously undergone ART procedures, including intrauterine insemination (IUI) and intracytoplasmic sperm injection (ICSI), which failed. The female partner was diagnosed with a thin endometrium (<7 mm) and underwent hysteroscopy, revealing no other significant intrauterine pathologies. Following hormonal treatment and ovum pick-up, hysteroscopic PRP was administered, resulting in improved endometrial thickness (ET) and successful embryo implantation, as evidenced by a positive serum β-hCG level of 1470 mIU/mL. This case demonstrates the hysteroscopic injection of PRP's potential for increasing endometrial receptivity and enhancing ART outcomes in women with RIF due to thin endometrium, making it a promising alternative to conventional therapies.

## Introduction

The innovations of assisted reproductive technology (ART) have provided new methods for detecting early miscarriages. Recent advancements in ART have enabled couples who were previously unable to conceive to achieve viable pregnancies, improving the outcomes for struggling couples who experience recurrent implantation failure (RIF) [1]. Approximately 10% of women treated via *in vitro* fertilization (IVF) experience RIF [2]. While there is no universally agreed-upon definition of RIF, the most commonly accepted definition is when a woman under age 40 fails to conceive after transferring at least four good-quality embryos in three fresh or frozen cycles [3,4]. RIF may occur due to anatomical anomalies in the maternal uterus, chronic endometritis, an unreceptive endometrium, antiphospholipid antibody syndrome, and several immunological factors [5]. When male factors, oxidative stress, poor embryo quality, anatomical anomalies, and hydrosalpinx are absent, RIF is probably caused by impaired endometrial function, including abnormal growth of the endometrium or reduced vascularization [1]. Endometrial receptivity is essential for effective embryo implantation [6]. The thin endometrium is characterized by a mid-luteal endometrial thickness (ET) of 7 mm or less and reduced endometrial receptivity, resulting in reduced embryo implantation, lower pregnancy rates, and poor outcomes from ART [7]. Routine 2D/3D ultrasonography (USG) is widely used to assess the thickness of the endometrium and vascularity.

Hysteroscopy is a common surgical procedure used to treat intrauterine pathologies. It involves inserting a hysteroscope into the endometrial cavity through the cervical canal using fluid distension media [8]. Hysteroscopy can detect unsuspected intrauterine lesions such as synechiae, septum, endometritis, and polyps in asymptomatic patients (40-50%) [9]. Uterine abnormalities are one of the leading causes of RIF, making hysteroscopic examination of the intrauterine cavity more common [10]. Using hysteroscopy while treating might enhance reproductive outcomes by treating intrauterine pathologies that may negatively impact implantation rates [11]. Hysteroscopy provides an accurate visual evaluation of the uterine cavity and cervical canal. It can also be used to treat intrauterine pathology immediately [10].

Platelet-rich plasma (PRP) preparation involves centrifugation of the patient's fresh peripheral blood to elevate the platelet concentration [12]. Platelets, with 2-3 μm diameter, are small cell fragments without nuclei produced by megakaryocytes in the bone marrow [13]. PRP is infused intrauterine. Several proteins, growth factors (GFs), and cytokines in the platelet act on the endometrium by promoting neo-angiogenesis and cell proliferation, as well as having anti-inflammatory qualities that lead to successful implantation [14]. PRP contains a variety of GFs, such as transforming growth factor (TGF), platelet-derived growth factor (PDGF), epidermal growth factor (EGF), vascular endothelial growth factor (VEGF), and other cytokines that stimulate proliferation and growth [15]. A family of secreted dimeric polypeptides known as PDGFs strongly induces the development of mesenchymal cells [16]. More precisely, PDGF signalling induces extracellular matrix production and remodelling, cytoskeletal flexibility, and migration of cells, all of which contribute to proliferation and angiogenesis [17]. An innovative approach for treating resistant endometrium is the hysteroscopic instillation of PRP in the sub-endometrial region [18]. Injecting autologous PRP into the uterus improves ET and has been shown to enhance implantation and clinical pregnancy rates in ART cycles compared to using hormone replacement therapy alone [16].

This case report aims to assess the hysteroscopic instillation of autologous PRP into the uterine cavity as an intervention for pregnancy outcomes in females who have been suffering from RIF.

## Case presentation

Patient information

A female patient aged 33, along with her partner aged 36, reported their issue with primary infertility after visiting a test tube baby centre in India in July 2023. They have been married for the past 11 years and have been unable to conceive since then. The couple attempted to achieve pregnancy for 10 years without using contraceptives. Initially, the couple tried to conceive naturally from 2015 to 2019. Neither of them had a habit of smoking, drinking, and using tobacco. The male patient did not exhibit any specific symptoms of infertility. The male's body mass index (BMI) was 23.6 kg/m^2^ and the female's was 22.5 kg/m^2^. The couple was provided with detailed information about all treatments, including their advantages and disadvantages, and they gave informed consent.

Clinical history

The couple had undergone intrauterine insemination (IUI) in February 2021 and ICSI failed cycle in August 2021, December 2021, and March 2022. RIF was also diagnosed in July 2022. Both partners had no previous history of asthma, heart disease, tuberculosis, or hypertension. The couple had no sexually transmitted diseases (STDs). There were no hereditary conditions found among close family members, and there was no known history of mental or psychiatric disorders.

Diagnostic assessments 

The husband's semen analysis revealed 8% normal morphology, 32% progressive motility, and a sperm count of 72 mil/mL. His report stated that his sperm profile was normozoospermia. The female partner went through particular investigations to determine the root cause of infertility. The investigations comprised the follicle-stimulating hormone (FSH) and anti-mullerian hormone (AMH) level tests, which were below the reference limit. Table 1 indicates the hormonal profile of the female patient.

**Table 1:** Hormonal profile of the female patient AMH: Anti-mullerian hormone; FSH: Follicle-stimulating hormone; LH: Luteinizing hormone

Hormonal Profile	Patient Value	Reference Value
AMH	0.6 ng/mL	0.8-1.0 ng/mL
FSH	8 IU/L	10 IU/L
LH	4 U/L	5 U/L

**Figure 1:**
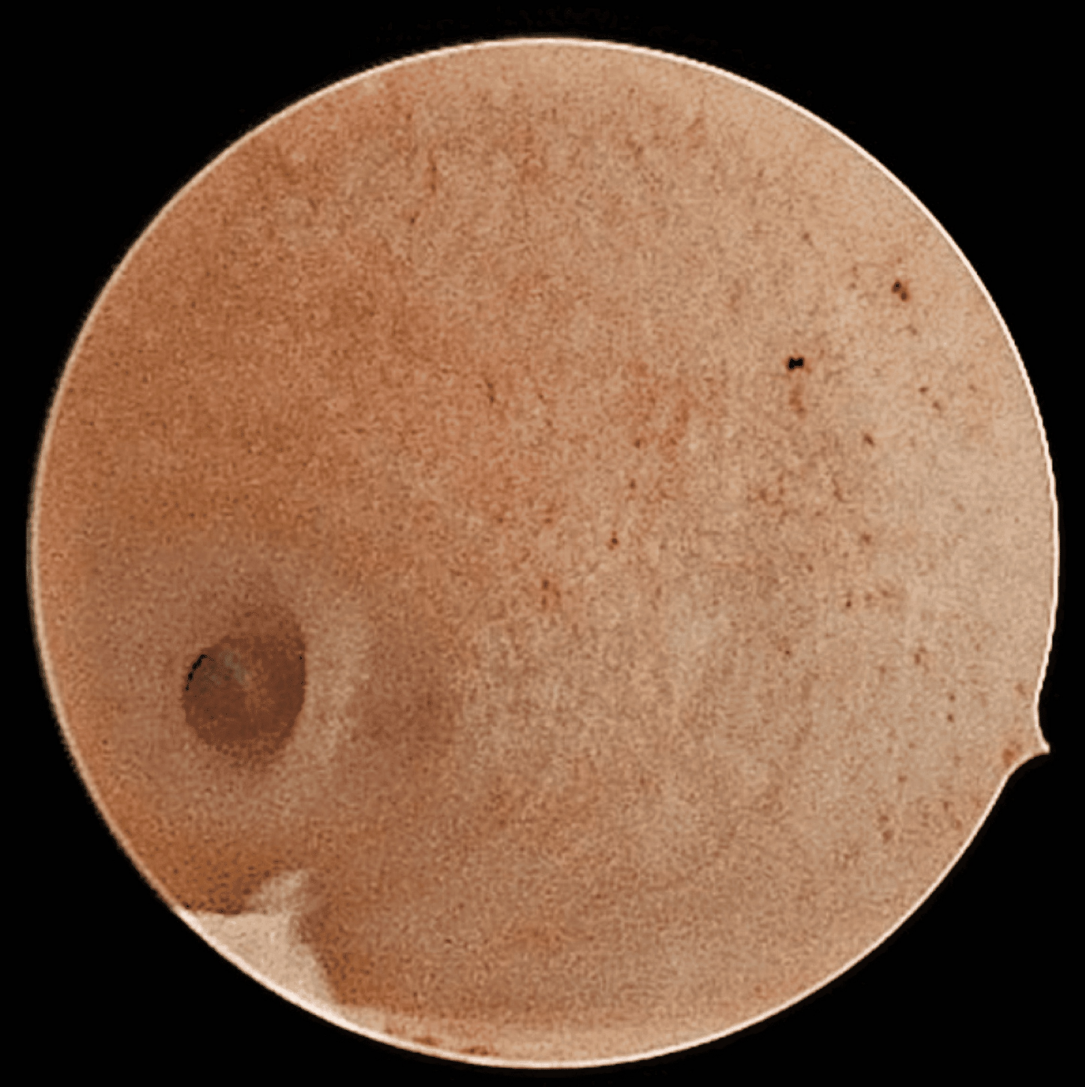
Hysteroscopic image indicating thin endometrium

Primary infertility indicated a chance of higher levels of reactive oxygen species (ROS) or other factors resulting in a thin endometrium, even though normal sperm parameters were present in the patient's husband. The female patient was diagnosed with a thin endometrium (<7 mm) and underwent a hysteroscopy (Figure 1). As a result, a thin endometrium was visible, which could be the factor of RIF.

Treatment

Gonadotropin-releasing hormone (GnRH) agonists and antagonists are medications used to regulate the timing of ovulation and stimulate the development of several follicles in the ovary. The female patient has received a trigger for oocyte pick-up. Following 36 hours of stimulation, an OPU procedure was planned.

During the procedure, four oocytes were extracted: two at metaphase II (MII), one at metaphase I (MI), and one at the germinal vesicle (GV) stage. ICSI was carried out on the same day, and the male patient was recommended to get a fresh semen sample. Following collection, the sample was processed and mature oocytes and good-quality sperm were used for ICSI. A good-quality cleavage stage developed.

On day 3, five embryos were cryopreserved. In the progressive cycle, hysteroscopic guided PRP was instilled on day 10, and the patient's consent was obtained prior to the procedure by providing detailed information about the advantages and disadvantages of the procedure. We added 1.5 mL of anticoagulant to each of two 15 mL test tubes and collected 13.5 mL of blood, maintaining a 1:9 ratio of anticoagulant to blood. The tubes were mixed by inversion and centrifuged at 1500 RPM for 15 minutes. We separated the plasma-rich platelets, ensuring at least 50% plasma volume without the buffy coat, and transferred the plasma to a new tube using a sterile pipette. After a second centrifuge at 3500 RPM for 15 minutes, we discarded most of the plasma, leaving 1 mL on top of the platelet pellet. The PRP was frozen in liquid nitrogen for a minute, then thawed by rolling it in the palm.

**Figure 2:**
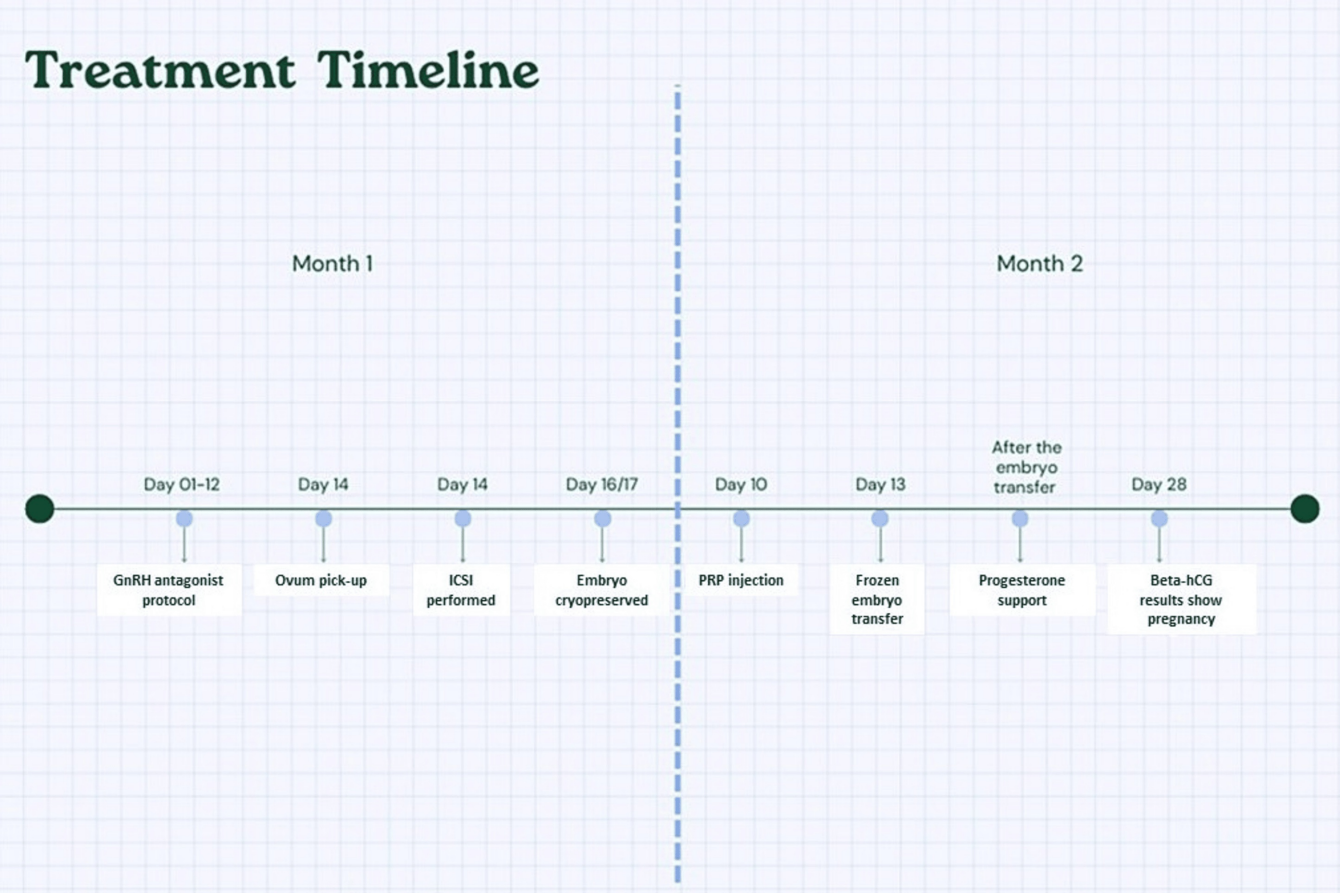
Treatment timeline of the patient from day 01 to day 28 of the progressive month ICSI: Intracytoplasmic sperm injection; PRP: Platelet-rich plasma; hCG: Human chorionic gonadotropin

Finally, we performed a third centrifuge at 3500 RPM for 10 minutes to remove platelet membrane debris or leave it as is. Intravenous anaesthesia was given to the female patient. 1 mL of autologous PRP was administered into the endometrium at a depth of 3 mm in four cardinal directions-the superior, inferior, left, and right sides of the uterine cavity-with 0.25 mL per direction under the guidance of hysteroscopy. After 2 days of PRP therapy, the endometrium thickness reached 7.5 mm. On day 13, two embryos of grade 4AA were transferred. The overall treatment timeline from Day 01 to Day 28 of the progressive month, including the procedures followed, is mentioned in Figure 2.

Follow up

After the embryo transfer, the patient was advised to continue her medicines, including 200 mcg of oral progesterone for 14 days, to enhance implantation and promote uterine lining growth. The patient received counselling on dietary and lifestyle advice, including recommendations for a balanced diet, regular exercise, and minimising potential risks. After 14 days of embryo implantation, the serum beta-human chorionic gonadotropin (β-hCG) level in the female blood sample was reported as 1470 mIU/mL.

## Discussion

Previous research indicates that among infertile women undergoing ART, 1.5% had <7 mm ET and 9.1% had <8 mm ET [19-21]. This case study indicates the challenges encountered by a couple diagnosed with RIF. The endometrium is crucial for the success of pregnancy rates. With its ability to enhance ET and receptivity, PRP implementation is recommended for infertile women with RIF or thin endometrium. Kumar et al. concluded that using PRP to promote the development of the endometrium and two PRP injections were recommended to induce sufficient endometrial development in patients with 7 mm ET, although PRP has been made up of autologous blood of the patient with low potential risk and safe without causing any immunological reactions and transmission of the infection [22,23].

A study by Agarwal et al. was performed on the hysteroscopic guided PRP installation on 32 patients, and the results included that all patients tolerated the PRP infusion well and experienced no adverse effects. In 24 out of 32 patients, ET was 7 mm or thicker in the next cycle followed up to day 15. In 4 out of 32 patients, ET was between 6 and 7 mm. In 4 of the 32 patients, ET did not increase and stayed below 6 mm. Because in these 8 patients, there was no increase in sub-endometrial blood flow and ultimately no improvement in the endometrial layer. In 28 of them, embryo transfer was carried out. Because the patients did not reach the ideal ET, 8 patients had their cycles cancelled. According to this study, the approach to enhancing ET through the hysteroscopic installation of PRP has achieved positive outcomes and opened up new possibilities for PRP application in thin endometrium cases [4,18,24,25].

During menstrual cycle days 11 to 13, hysteroscopic injections were administered. Following the PRP injection, ET increased in 55.3% of the patients, qualifying them for embryo transfer within the same cycle [21]. Treatments for thin endometrium have also included vitamin E, which increases capillary blood flow in various organs, and sildenafil citrate, which increases blood flow in the uterine artery [5,26-28].

Pounikar et al. reported that approximately 40% of intrauterine pathologies were undetected by other standard techniques like USG or hysterosalpingography (HSG), but hysteroscopy could detect and observe them. The IVF success rate after hysteroscopy showed a statistically significant improvement (30% vs. 23.3%) [9].

Patel et al. discussed the possibility of adverse effects and risks of PRP therapy. Patients often experience localized adverse reactions at the injection site, such as pain, swelling, and bruising, which are usually transient and subside within a few days. PRP therapy, particularly through injections, poses a potential risk of infection, making it crucial to maintain strict aseptic techniques and sterility during its preparation and administration to minimise this risk.

The need for standardized protocols and guidelines for preparing and administering PRP is a significant challenge. Standardizing PRP preparation methods and selecting the most appropriate technique for the specific clinical application is crucial for achieving consistent and predictable treatment outcomes. Patient adherence to regular exercise, a balanced diet, and avoiding harmful factors such as smoking or excessive alcohol can significantly enhance the efficacy of PRP therapy, leading to more favourable treatment outcomes [29-31].

Advanced diagnostic techniques like hysteroscopy, combined with innovative therapies like PRP, can considerably enhance results for couples dealing with RIF. PRP therapy has the potential to be a beneficial intervention for patients with RIF and thin endometrium, as demonstrated by the positive outcome in this case. Compared to conventional hormonal therapies, which might not always be successful, it presents an effective alternative [32]. To improve outcomes, future studies should include a larger sample size and standardized protocols for hysteroscopic PRP administration while acknowledging the limitation of the study’s single-case design, which may not generalize to a broader population.

## Conclusions

The effects of combining hysteroscopy with PRP therapy on enhancing endometrial receptivity and improving pregnancy outcomes in RIF patients were demonstrated. Hysteroscopic PRP instillation seems to be a safe and efficient method to treat thin endometrium and improve ART outcomes. The administration of PRP, with a significant level of growth factors promoting neo-angiogenesis and proliferation of cells, addresses the challenge of thin endometrium by improving endometrial receptivity. Furthermore, studies and research are required to develop standard protocols and ensure the long-term safety and effectiveness of this innovative method.
